# Cost of behavior change communication channels of *Manoshi* -a maternal, neonatal and child health (MNCH) program in urban slums of Dhaka, Bangladesh

**DOI:** 10.1186/1478-7547-11-28

**Published:** 2013-11-14

**Authors:** Bidhan Krishna Sarker, Sayem Ahmed, Noushin Islam, Jahangir AM Khan

**Affiliations:** 1 Centre for Reproductive Health, icddr,b, GPO Box 128, Dhaka 1000, Bangladesh; 2 Health Economics & Financing Research Group, Centre for Equity and Health System; icddr,b, GPO Box 128, Dhaka 1000, Bangladesh; 3 Health Economics Unit, Department of Learning, Informatics, Management and Ethics, Karolinska Institute, Stockholm, Sweden

**Keywords:** Cost, BCC, MNCH-intervention, Micro-costing, Urban-Slum, Dhaka-Bangladesh

## Abstract

**Background:**

The cost of behavior change communication (BCC) interventions has not been rigorously studied in Bangladesh. This study was conducted to assess the implementation costs of a BCC intervention in a maternal, neonatal and child health program (*Manoshi*) run by BRAC, which has been operating in the urban slums of Dhaka since 2007. The study estimates the costs of BCC tools per exposure among the different types of BCC channels: face-to-face, group counseling, and mass media.

**Methods:**

The study was conducted from November 2010 to April 2011 in the Dhaka urban slum area. A micro-costing approach was applied using primary and secondary data sources to estimate the cost of BCC tools. Primary data were collected through interviews with service-providers and managers from the *Manoshi* program, observations of group counseling, and mass media events.

**Results:**

Per exposure, the cost of face-to-face counseling was found to be 3.08 BDT during pregnancy detection, 3.11 BDT during pregnancy confirmation, 12.42 BDT during antenatal care, 18.96 BDT during delivery care and 22.65 BDT during post-natal care. The cost per exposure of group counseling was 22.71 BDT (95% CI 21.30-24.87) for Expected Date of Delivery (EDD) meetings, 14.25 BDT (95% CI 12.37-16.12) for Women Support Group meetings, 17.83 BDT (95% CI 14.90-20.77) for MNCH committee meetings and 6.62 BDT (95% CI 5.99-7.26) for spouse forum meetings. We found the cost per exposure for mass media interventions was 9.54 BDT (95% CI 7.30-12.53) for folk songs, 26.39 BDT (95% CI 23.26-32.56) for street dramas, 0.39 BDT for TV-broadcasting and 7.87 BDT for billboards. Considering all components reaching the target audience under each broader type of channel, the total cost per exposure was found to be 60.22 BDT (0.82 USD) for face-to-face counseling, 61.40 BDT (0.82 USD) for group counseling and 44.19 BDT (0.61 USD) for mass media.

**Conclusions:**

The total cost for group counseling was the highest per exposure, followed by face-to-face counseling and mass media. The cost per exposure varied substantially across BCC channels due to differences in cost drivers such as personnel, materials and refreshments. The cost per exposure can be valuable for planning and resource allocation related to the implementation of BCC interventions in low resource settings.

## Background

Maternal, neonatal and child health (MNCH) services are of high importance in Bangladesh for meeting millennium development goals (MDG) 4 and 5. Services under the MNCH banner include antenatal care (ANC), delivery care, post-natal care (PNC), newborn care, and child health care. Behavior change communication (BCC) refers to the delivery of communication strategies that aim to promote positive behaviors appropriate to the local settings, and are therefore considered an integral part of MNCH services. Costing is a major activity used to define future service packages and determine the funding of activities in the healthcare system [1]. The costs of selected services for several MNCH programs have previously been calculated [2-4]. However, costing of BCC interventions have not been rigorously conducted in Bangladesh, and the cost of individual components of BCC during ANC, PNC, delivery, and counseling have not been considered separately [5].

Over the last few decades, a large number of MNCH programs have been initiated in Bangladesh targeting the rural and urban poor population. *Manoshi*, a MNCH program run by BRAC (formerly Bangladesh Rural Advancement Committee), has been implemented since 2007 within urban slums in Bangladesh. BCC interventions have been an important component of this program. The services of *Manoshi* are free to consumers, meaning that sustainability is a potential challenge. Information about the cost of providing specific services of *Manoshi*, such as BCCs, medical care, etc. is thus important for designing future programs as well as making refinements to the current program. Furthermore, several previous studies lack the costing of services at a low level of disaggregation, such as the cost of individual and group counseling [6,7]. This current study aimed to address this gap in the evidence by calculating the cost of BCC interventions for specific components of the *Manoshi* program in Bangladesh to a cost-per-exposure level.

### BCC intervention in Manoshi program

BRAC, a leading NGO of Bangladesh, had been delivering MNCH services through a community-based health intervention, under the *Manoshi* program, in selected urban slums of all city-corporation areas in Bangladesh. This program aims to reduce maternal and child mortality in urban slums. BCC interventions are a key component of *Manoshi,* with the goal of developing awareness about MNCH in the target audience. The development of BCC messages and identification of message delivery channels was undertaken by BCC experts within BRAC, who work closely with the urban slum community. The effectiveness of *Manoshi’s* BCC messages was demonstrated in recent study, which found that several BCC tools were associated with significant increases in MNCH knowledge: specifically, group counseling, TV spots, leaflets, sticker increased knowledge among exposed women by 2.3, 2.8, 3.8 and 2.3 times respectively in comparison with non-exposed women [8]. In a cost-effectiveness study in Bangladesh, Hutchinson *et al*. found similar BCC tools (TV spot, billboards, a drama serial, IEC print material, press and other media campaigns) were effective family planning interventions [6]. Infant and young child feeding practice programs in Bangladesh have applied similar BCC tools for improving mother’s knowledge of exclusive breastfeeding and nutrition [9]. A randomized-control study in the Philippines found that a 6.4% increase in contraceptive use could be attributed to national mass media campaign [7].

The target audiences of BCC interventions are new couples (newly married women and their husbands), eligible couples (married women of reproductive aged 15–49 years having at least one pregnancy experience, and their husbands), pregnant women and their husband, lactating mothers and their family members, and the broader community. The community health volunteers *Shasthya Sebika* (SS), community health workers *Shasthya Karmi* (SK) and Program Organizers (PO) are directly involved with delivering BCC interventions. The disseminated key BCC messages are as follows:

• At least 3 antenatal check-ups are needed during pregnancy;

• Eat more food and drink more water and liquids during pregnancy;

• Keep a sound mind and take proper rest during pregnancy;

• Ensure that deliveries take place at a delivery centre (birthing hut);

• Use proper family planning methods after 42 days of delivery of the baby;

• Use clean pad after delivery and keep the birth passage dry and clean;

• Family members should take care of pregnant and lactating mothers;

• Inform BRAC health workers immediately if any danger signs develop with the mother;

• Wrap the whole body of the baby with clean cloths and always keep the baby warm;

• Initiate colostrums feeding;

• Keep the umbilical stump dry and bathe the newborn at least after seven days from birth;

• Inform BRAC health workers immediately if there are any danger signs of the neonate;

• Ensure exclusive breast feeding for up to six months;

• Start complementary feeding from the age of 7 months;

• Immunize the baby against seven infectious diseases;

• Use oral re-hydration solution (ORS) for diarrhea;

• If the baby has rapid breathing, chest in-drawing, respiratory problem or any other danger signs, inform a BRAC health worker immediately;

Additionally, messages outlining maternal and child health danger signs were also disseminated to increase awareness in the community. These included signs of excessive bleeding and convulsions during pregnancy, and rapid breathing, lethargy, convulsion for children.

BCC messages were delivered through 13 different mediums, which can be categorized into three broader channels, namely, face-to-face counseling (FFC), group counseling (GC) and mass media (MM). The following section explains how those three broader channels and their sub-channels functioned for message dissemination.

#### Face-to-face counseling (FFC)

This channel included five sub-channels for the delivery of BCC messages, including: pregnancy identification; pregnancy confirmation; antenatal care visits; delivery care at birthing huts; and post-natal care visits. Community Health Workers provided FFC to the target audiences with selected BCC messages during their regular field visits and when providing MNCH services. Messages focused on pregnancy, safe delivery, and neonatal and child health issues.

#### Group counseling (GC)

This channel included four sub-channels, including: EDD (expected date of delivery) meetings for women who were close to their expected delivery; women’s support group meetings (WSG); maternal, neonatal and child health meeting (MNCH); and spouse forum meetings. The program formed these groups with different target groups within the community, i.e. 7–9 month pregnant women for EDD meetings; prominent community women for WSG meetings, influential members of the community for MNCH meetings, and pregnant women together with their husbands for spouse forum meetings. Through these joint efforts, the program aimed to build awareness of safe delivery, birth preparedness, and birthing huts (safe delivery centres) to the community.

#### Mass media (MM)

This channel included four sub-channels: folk song performance; street dramas, TV spots; and billboards. The program hired staff with expertise in developing and delivering cultural tools, such as folk singing and street dramas, to oversee this area of the intervention. The BCC messages were provided through artists singing folk songs and performing dramas in public places. These events were usually organized between October and April to avoid the rainy season, and took place at least once per year in each BRAC delivery centre (birthing hut). A one minute TV spot was broadcast to the community through the video channels of a local cable network. Usually, it was shown 10–12 times each day during the broadcasting of movies. The billboards for the program were set up in highly visible areas beside major streets in the city, and featured informational messages relating to maternal and child health.

We accounted for the variation between BCC channels by listing and defining the different inputs required for their operation. For instance, FFC involves community health workers and volunteers, while TV spots require the involvement of cable television service providers. Moreover, different channels might reach audiences on different scales. For example, while FFC reached a smaller audience, MM covered a larger population. The cost per exposure therefore needs to take into account the audience reached by each BCC channel or sub-channel.

## Methods

The methods that were used to calculate the cost of BCC channels and sub-channels of the *Manoshi* intervention are outlined in the following section.

### Costing approach

For estimating costs of BCC channels used in the *Manoshi* project, the following assumptions have been made:

1) The BCC channels are effective;

2) Implementation costs are included;

3) The cost of BCC channels is calculated from the provider’s perspective

### Micro-costing

The study used a 'micro-costing’ method for calculating the cost of inputs for the *Manoshi* BCC interventions. Micro-costing is a meticulous, bottom-up and highly detailed approach [2]. It is used when the accuracy of resource measurement is crucial and data collection to the required detail is feasible and affordable [10]. Information on the inputs and costs were available from the implementing organization (BRAC) and the market values were used whenever applicable. The following equation was applied for calculating the cost of each sub-channel of the BCC intervention [11].

$$\begin{array}{ll} \text{Cost}\;\text{of}\;\text{BCC}\;\text{intervention}= & \sum \;\left(\text{Quantity}\;\text{of}\;\text{any}\;\text{input}\right)\\ & \left(\times \text{unit}\;\text{price}\;\text{of}\;\text{that}\;\text{input}\right) \end{array}$$

For example, to estimate the cost of FFC sessions, the inputs required (e.g. staff time) and quantity of the inputs was identified. For staff time, unit price (per hour salary) was calculated from the monthly salary. Cost for staff time was then calculated by multiplying the number of hours devoted by staff to running FFC sessions with the per hour salary.

#### Calculation of cost per BCC exposure

To calculate the cost per exposure for each BCC sub-channel, the total cost of each BCC sub-channel was divided by the estimated number of participants or people targeted by that specific channel. Number of participants with direct contact (or for mass media, the people targeted) of each sub-channel was estimated by direct observation undertaken by the research team members and available secondary data. Cost per exposed person for each channel was thus calculated by adding up the costs per exposed for all sub-channels within one category.

### Cost components of the program

The cost items were classified into “recurrent cost items” and “capital cost items”. Discounting was used to incorporate time differentials. These concepts are described further in the following section.

#### Recurrent cost

Regular costs incurred repeatedly for any BCC service within one year was considered a “recurrent cost”. BCC materials, refreshments, salary of BRAC personnel, etc. are examples of recurrent cost items. Unit costs of recurrent items were collected by interviewing BRAC personnel who were involved in supplying those items. The prices were then cross-checked with field staff. For example, refreshments provided during GC cost 15 BDT for each participant. A portion of salary was assigned to BCC activities for personnel who were actively participating in arrangement of BCC counseling sessions. Furthermore the cost of training staff in delivering the intervention was included in calculations. However, the training cost of supervisors who generally give a negligible portion of his/her time to these activities was not included in the total cost.

#### Capital cost

According to Drummond *et al*. [11], capital costs or assets are usually invested in a bulk amount and used over time. Capital items such as fans, clocks, cabinets, mats, boards, curtains, register books and sign boards had been used to equip the venue for counseling interventions. Different life times were assumed for different capital items. For instance, a five year life time was considered for ceiling fans, cabinets, etc. Life times for clocks and sign boards were considered to be two years and one year respectively.

#### Discounting and annuitization

Costs should be discounted to their present value to adjust for any time differentials [11,12]. Capital items were annualized for their respective functional lifetime. In absence of any official guidelines on discount rates applicable in Bangladesh, this study has used 3% discount rate for annuitizing as suggested by a number of guidelines [13,14]. Using this discounting rate, cost per year of every capital items was calculated using the following equation:

$$E=K{\left(1+\frac{1}{\left(1+r\right)}+\frac{1}{{\left(1+r\right)}^{2}}+\cdots \right)}^{-1}$$

Where, K = Purchasing price, r =  Discounting rate and E = Equivalent yearly cost.

When calculating the costs of staff training, a 54-month project period was applied since the BCC intervention started in July 2007 (6 months later than the start of *Manoshi* program period) and continued until the end of 2011. Inflation and discounting had been applied whenever applicable.

#### Fixed and variable costs

The cost components of the program can be separated into fixed and variable costs. The costs that did not change with an increase in the number of persons exposed by BCC were considered to be fixed costs, while costs that increased with exposure to more individuals were considered to be variable costs.

Information on all recurrent costs were collected during study period (November, 2010 to April, 2011). The items which were purchased in earlier years, we made adjustments for those to the value of year 2011 and then summed up with other recurrent/capital costs. All costs are reported for 2011’s value.

### Inputs in BCC interventions

The inputs generally used in BCC interventions were staff time, BCC materials, logistics and supplies, supervision, capital items, and products contracted to external organizations. We considered different inputs to calculate the cost of different channels. For the Face to Face Counseling channel, inputs included staff time (community health workers), staff training, logistics and supplies, BCC materials (posters, stickers), supervision (PO), and venue rent. For the Group Counseling channel, inputs included staff time (SK, PO, MMW, BM), staff training, logistics and supplies, BCC materials (poster, sticker, flip chart), supervision, refreshments, capital items (e.g. table, chair, clock, stethoscope, umbrella), and rent of birthing huts (delivery centres). For the Mass Media channel, inputs included: the folksong team (contracted out) and supplies for the folk song activities (e.g. banners, leaflets, badge, rope); the street drama team (contracted out) and supplies for the street drama activities (e.g. leaflets, communication for street drama); production of the TV spot, CD, broadcasting fees, and communication with the TV station; and billboard production and set up (contracted out).

Ten minutes were allocated to BCC message delivery during all sub-channels in FFC except pregnancy identification, which was allocated five minutes. However, the total time for these events ranged from five to 24 minutes. The staff time was transformed into costs using the average salary of the relevant staff.

Time allocated to BCC message delivery was generally higher in GC sub-channels. The duration of EDD meetings was 90 minutes, and half of this time period was devoted to BCC message delivery. Duration of each of WSG and MNCH meetings was 45 minutes, of which 15 minutes were allocated for BCC message delivery. A Spouse Forum meeting lasted for 45 minutes, where 10 minutes went to BCC message delivery. Staffs were also involved in communicating to prospective mothers, their husbands or relatives and community people so that they could attend the meeting.

Most of the activities in MM were contracted out to external organizations. However, some of the activities, such as communication with external organizations and event coordination activities, required time from BRAC staff and thus were included in the cost calculations. BRAC hired both folksong and street drama team for conducting the sessions. Additional supplies not provided by the contracted teams, such as leaflets distributed to audiences during performances, and props (like banners, badge and rope) for performances, were also included in calculations. An average of 150 and 200 people attended the folksong and street drama sessions respectively.

The TV spot (one minute advertisement) was produced by an advertising firm and broadcasted through the video channels of a local satellite cable connection. The production cost was a one-off payment directly to the advertising firm, while the broadcasting fees were paid monthly to the cable connection providers for a period of 54 months. The size of the exposed audience was estimated using data from the *Manoshi* regional office, available demographic data [15], and estimates of the proportion expected to be exposed based on previous research into the *Manoshi* program. Based on the national demographic structure, the total population within the study area was estimated to be 3,250,592, of which 68 percent were considered to be adults (2,210,403), and half of them were female [15]. Sixty nine percent of adult females (762,589) and 30 percent of adult males (331,560) were assumed to be exposed to the TV spot over the course of one year, based on the findings from another study done by Sarker *et al.,*[8]. Therefore, in total 182,358 adults were estimated to be exposed to the TV spot in six month period. The total cost of the TV spot was divided by the exposed audience when calculating the cost per exposure.

Billboard production and setup was contracted to an external agency for an annual fee. Twelve billboards were set up in different areas of Dhaka. The total population of those areas was 112,920, of which 68 percent were expected to be adults. Previous research into billboard exposure for *anti-smoking* program found that 12.3 percent of women and 20.8 percent of men were exposed to billboards during a one month period [16]. Applying the same exposure rate in this current study, in total 12,708 adults were estimated to be exposed to the billboard in one month period.

### Data collection

The study was conducted from November 2010 to April 2011, with all cost information collected during this period. Data on inputs and prices were collected from both primary and secondary sources. Primary data were obtained by interviewing BRAC staffs who were involved in implementing BCC tools. Data on contractual agreements, training costs, salaries, capital items and related costs were collected from the primary source. We observed different types of group counseling sessions and Mass Media sessions to document the duration of sessions and the time allocated for BCC message delivery, use of BCC materials, time commitment for various types of staff, number of participants, and the capital items used. To document the group counseling sessions, we carried out five observations for each of the WSG meetings, spouse forum meetings, and MNCH meetings, and 10 observations of the EDD meetings. In addition, we observed four folksong and nine street drama sessions in the field. A total of 38 observations were made to document the GC and MM sessions. Details about the data collection observation activities are presented in Table 1. These sessions were undertaken in all five broad intervention areas of *Manoshi,* namely the Dhanmondi, Gulshan, Uttara, Mohammadpur and Jatrabari regions in Dhaka city. Secondary data collection included reviewing documentation of SS training costs and their performance records (such as the number of pregnant cases identified, ANC sessions, PNC sessions, and deliveries) that had been recorded in earlier studies of the *Manoshi* project [17,18]. For validation, information was verified with the head-office as well as staff at local project sites.

**Table 1:** Number of BCC sessions observed for cost data collection

**Counseling and mass media sessions**	**Number observed**
Women support group (WSG) meeting	5
MNCH meeting	5
EDD meeting	10
Spouse forum meeting	5
Street drama	9
Folk song	4
**Total**	**38**

## Results

This section presents the total cost per exposure through BCC channels under the three broad categories, as well as their sub-categories.

### Cost of face-to-face counseling (FFC)

The cost per exposed through the FFC category is presented in Table 2. The cost per exposure for a pregnancy identification event was 3.08 BDT. Monthly fixed and variable costs per birthing hut were calculated to be 38.95 BDT and 81.25 BDT. Since *SS* staffs were responsible for pregnancy identification, the cost of *SS* training (36.00 BDT) and salary (50.00 BDT) were included, as was logistics and supplies (2.95 BDT). Furthermore, the cost (31.25 BDT) for time allocated by the PO who supervised FFC services once a month was incorporated into the total cost.

**Table 2:** Cost per exposed person of sub-channels in face-to-face counseling

**Cost components**	**Inputs**		**Face-to-face sessions**				
			**Pregnancy identification**	**Pregnancy confirmation**	**ANC**	**Delivery**	**PNC**
Fixed cost item	Staff training	SS	36.00			22.16	
		SK		18.46	18.46	18.46	18.46
	Logistic & supply	SS	2.95			1.82	
		SK		7.59	7.59	7.59	7.59
	Venue					8.33	
Total fixed cost			38.95	26.06	26.05	58.36	26.05
Variable cost item	BCC materials	Poster			64.92		18.55
		Sticker			29.21		8.35
	Personnel	SS	50.00			49.23	
		SK		60.90	22.44	12.82	6.41
	Supervision	PO	31.25	31.25	31.25	31.25	31.25
Total variable cost			81.25	92.15	147.82	93.30	64.56
**Total cost**			**120.20**	**118.20**	**173.87**	**151.66**	**90.61**
Number of exposed per birthing hut			39	38	14	8	4
**Total cost per exposed person (BDT)**			**3.08**	**3.11**	**12.42**	**18.96**	**22.65**
**(US$**^**1)**^**)**			**(0.04)**	**(0.04)**	**(0.17)**	**(0.26)**	**(0.31)**

Note. ^1)^One US dollar was equivalent to 73 BDT at the time of the study.

Cost per BCC exposure during a pregnancy confirmation was estimated to be 3.11 BDT, and exposure for BCC during each antenatal and postnatal visit was estimated to be 12.42 BDT and 22.65 BDT respectively. During delivery of a pregnant woman, as *SS* and *SK* both were expected to be present, thus their associated costs were incorporated into calculations. The BCC cost per exposure during delivery was calculated to be 18.96 BDT. If a mother was exposed to all recommended services (including 3 ANC and 2 PNC visits), the estimated total cost per exposed was 87.54 BDT (US$ 1.20). It should be noted that the BCC materials were distributed only once per exposed during ANC and PNC periods.

### Cost of group counseling (GC)

The total cost for EDD, WSG, MNCH and spouse forum meeting were calculated at 272.55 BDT (95% CI 258.64-281.61), 113.99 BDT (95% CI 105.22-122.75), 142.66 BDT (95% CI 132.19-153.14) and 66.23 BDT (95% CI 58.27-74.19) respectively (Table 3). The number of participants was 12, 8, 8 and 10 at each meeting respectively. The cost per person exposed was thus calculated to be 22.71 BDT (95% CI 21.30-24.87), 14.25 BDT (95% CI 12.37-16.12), 17.83 BDT (95% CI 14.90-20.77) and 6.62 BDT (95% CI 5.99-7.26) for EDD meeting, WSG meeting, MNCH meeting and spouse forum meeting respectively. Among all GC sessions, the highest cost per session was observed for the EDD meeting, while the lowest was the spouse forum meeting.

**Table 3:** Cost per exposed person of sub-channels in group counseling

**Cost components**	**Inputs**		**Group sessions**			
			**EDD**	**WSG**	**MNCH**	**SF**
Fixed cost item	Staff training	SS	0.20	0.07		
		SK	0.13	0.04	0.04	0.03
		MMW	0.71			
		PO	0.22	0.07	0.07	0.02
		BM	0.10	0.03	0.01	0.01
	Capital		0.93	0.31	0.31	0.21
	Venue rental		4.17	1.39	1.39	0.93
Total fixed cost			6.46	1.83	1.83	1.20
Variable cost item	BCC materials	Poster	0.58	0.58	1.16	0.58
		Sticker	0.41	0.41	0.81	
		Flip chart	25.76	25.76	51.52	
	Personnel	SK	7.21	2.40	2.40	1.60
		PO	43.27	14.42	14.42	4.81
		MMW	27.04			
		BM	6.01	6.01	18.03	6.01
	Refreshment		150.00	60.00	50.00	50.00
	Communication	SS	5.15	2.06	2.06	1.60
		SK	0.64	0.43	0.43	0.43
Total variable cost			266.07	112.07	140.84	65.03
**Total cost for BCC per session (CI)**			**272.55 (258.64-281.61)**	**113.99 (105.22-122.75)**	**142.66 (132.19-153.14)**	**66.23 (58.27-74.19)**
**Number of participants in a session (CI)**			12 (10.27-13.13)	8 (6.36-9.64)	8 (6.04-9.96)	10 (7.77-12.23)
**Cost per exposed person (BDT)**			**22.71**	**14.25**	**17.83**	**6.62**
**(CI)**			**(21.30-24.87)**	**(12.37-16.12)**	**(14.90-20.77)**	**(5.99-7.26)**
**(US$)**			**(0.31)**	**(0.20)**	**(0.24)**	**(0.09)**

### Cost for mass media (MM) campaigning

The total cost for folksong performances and street drama presentations was calculated at 1,431 BDT (95% CI 1402.72-1459.33) and 5,277 BDT (95% CI 5251.01-5303.29) respectively (Table 4). The audience size was 150 and 200 attendees at each session respectively. The cost per person exposed was thus calculated to be 9.54 BDT (95% CI 7.30-12.53) and 26.39 BDT (95% CI 23.26-32.56) for folksong performances and street drama performances respectively. The TV spot was estimated to cost 70,450 BDT in total, and given 182,358 individuals were expected to be exposed to this channel, the cost per exposed was calculated at 0.39 BDT. The cost of billboards was found to be 100,053 BDT in total, and given the exposed population was expected to be 12,708 individuals, cost per exposed person was 7.87 BDT. A summary of the cost per exposure of different Mass Media channels is presented in the Table 4.

**Table 4:** Cost per exposed person for sub-channels in mass Media sessions

**Cost components**	**Inputs**		**Mass media**			
			**Folksong**	**Street drama**	**TV-spot**	**Billboard**
Fixed cost item	Hiring/Contract out		1,200.00^1)^	5,000.00^1)^	70,000.00^2)^	100,000.00^3)^
	Production cost			10.60	397.59	
	Arrangement items	Banner	1.43			
		Badge	3.43			
		Rope	0.19			
Total fixed cost			125.06	5010.60	70397.59	100,000.00
Variable cost item	BCC materials	Leaflet	121.72	162.30		
		SS	7.69	7.69		
		SK	4.81	4.81		
	Personnel	PO	28.85	28.85		
		MMW	18.03	18.03		
		BM	36.06	36.06		
		RM	8.81	8.81	52.88	52.88
Total variable cost			225.97	266.55	52.88	52.88
**Total cost**			**1431.03**	**5277.15**	**70450.47**	**10,0052.88**
**(CI)**			**(1402.72-1459.33)**	**(5251.01-5303.29)**	**-**	**-**
Total no of participants			150	200	182,358	12,708
**Cost per exposed person (BDT)**			**9.54**	**26.39**	**0.39**	**7.87**
**(CI)**			**(7.30-12.53)**	**(23.26-32.56)**	**-**	**-**
**(US$)**			**(0.13)**	**(0.36)**	**(0.01)**	**(0.11)**

^1)^Per show, ^2)^Broad casting through cable TV, ^3)^Display per year.

In summary, the total cost per exposed was the sum of sub-channels within each of the three broader categories of BCC channels. The highest cost per exposed was observed in GC, followed by FFC and MM (Table 5). These costs were applicable if an individual was exposed to each and every sub-channel within the respective broad category of channels.

**Table 5:** Total cost per exposed person in different BCC channels (in BDT and US$)

**BCC channel**	**Total cost per exposed**	
	**BDT**	**US$**
Face-to-face counseling	60.22	0.82
Group counseling	61.40	0.85
Mass media	44.19	0.61

## Discussion

Among the three broader channels, FFC was considered the primary type of intervention, through which most of the target audience (pregnant women and mothers) in the *Manoshi* program were exposed. All five sub-channels within FFC were essential for exposure to the target audience, and any single sub-channel within FFC was not considered to be a complete BCC intervention on its own. It is, therefore, important to notice that FFC was considered to be a complete set and the total cost of FFC should be considered in economic planning of an MNCH program. The total average cost per exposure was estimated to be 87.54 BDT (US$ 1.20), which included one event of pregnancy identification (3.08 BDT) and confirmation (3.11 BDT), three ANC visits (23.81 BDT each), one delivery care at *Manoshi* birthing hut (18.96 BDT) and two PNC visits (38.58 BDT each). The BCC materials were distributed only once per exposed during ANC and PNC visits. Considering the total FFC cost per exposure, personnel costs were the highest (30.83%), followed by supervision (23.87%), staff training (20.17%), BCC materials (18.49%) logistics and supplies (5.37%) and venue rent (1.27%). It’s worth noting that low-cost personnel were involved in delivering FFC sessions, which kept the overall cost relatively low.

Among the FFC sub-channels, we found PNC visits had the highest cost per exposure. PNC was generally received by a fewer number of mothers compared to other intervention, which made the cost relatively high. Next, BCC during delivery was found to be next most expensive FFC session, which was attributed to the longer involvement of SS personnel during delivery, as BCC messages are given (along with other services) during this time. The cost of BCC during ANC was predominantly attributed to materials, such as posters and stickers. Finally, the cost for BCC messages during pregnancy identification was low since the time devoted to delivering BCC messages was very short in this sub-channel.

In the group counseling sessions, almost identical messages were given to prospective mothers, their husbands and the community as during the FFC sessions. Group counseling was thus considered a complementary intervention in the sense that it reminded the prospective mothers about the BCC information delivered during pregnancy identification, confirmation, ANCs and PNCs. In addition, husbands and relatives received BCC messages which would have increased awareness in the people immediately surrounding the pregnant women. The highest cost per exposed was observed in EDD meetings (22.71 BDT), followed by MNCH (17.83 BDT), WSG (14.25 BDT) and spouse forum meetings (6.62BDT).

The highest cost of GC was for EDD meetings, explained by the involvement of relatively higher salaried personnel for a 90 minute session, of which half was spent delivering BCC information. WSG and MNCH meetings were similar a number of ways, including number of participants, staff involvement and duration of the BCC message delivery. The difference in cost between WSG and MNCH meetings can be explained by the differential involvement of higher salaried personnel and a lower frequency of meetings for the later type, with WSG meetings held every month while MNCH meetings were held every two months. The cost of spouse forum meetings was smallest among all GC sessions because of fewer materials were used and the events were shorter in duration.

Among the four types of Mass Media sub-channels, the highest cost was street drama presentations (26.39 BDT), followed by folksong performances (9.54 BDT), billboards (7.87 BDT) and TV spots (0.39 BDT). Folksong performances and street drama presentations were by nature targeting a specific group, while TV spots and billboards reached a broader audience. While the former two sub-channels’ cost was higher due to the lower possibility of exposure, the latter two cost far less due to exposure to larger population. Folksong performances were less expensive than the street dramas due to differences in the price of hiring the external teams (1,200 BDT vs 5,000 BDT respectively).

This study intended to consider costs at the implementation level, which means the cost of overseeing the project at higher levels (such as central management, planning) was not included. However, the implementation personnel gave the greatest proportion of time to the BCC intervention. Therefore, the cost of upper-level staff time was not expected to contribute greatly to overall BCC costs.

We have presented the total costs of the BCC intervention separated into fixed and variable costs, since a low variable cost means that a higher number of exposures results in a lower total variable cost. In addition, with higher number of exposures, the fixed cost will be divided by a higher number of exposed individuals, which correspondingly reduces the total cost per exposure.

A limitation of this study is that it is not an analysis of costs from the societal perspective. It instead focused on the costs of providing BCC services through different channels. For instance, no costs for households or individuals receiving BCC (such as time spent attending group counseling) is not included in this analysis. Further, because of the nature of data collection, we are unable to calculate confidence intervals (CI) or uncertainly for other channels (FFC and MM). For FFC, we took data from program staffs on the basis of consensus among themselves through a qualitative manner. It means that the BRAC staffs reported only one figure about any inputs used in FFC. For MM, we observed several events only for 2 sub-channels (street drama and Folk songs) and we therefore, are able to report CI on these items only.

An impact evaluation study of *Manoshi* project was conducted in 2007, 2009 and 2011 [19]. These studies assessed the impact of BCC channels on women’s knowledge of services required during pregnancy, during delivery, after delivery and when caring for children, in a project area and a comparison area. An overall improvement in knowledge about services (ANC, PNC and delivery), and possible complications during pregnancy and for newborn, was found. Specifically, over a four year period, knowledge about the importance of attending four ANC visits increased from 44.7% to 58.7%, while awareness of danger signs during pregnancy (such as high fever) increased from 16.0% to 32.2%. Improvement in knowledge was also observed for PNC services, newborn, and child health care. However, this study could not determine what delivery channels were responsible for the changes. , the person exposed that we used as denominator of cost calculation is not homogeneous in its effects.

The calculation of BCC interventions at such a disaggregated level was not found in other studies in Bangladesh. It was thus first of its kind. A number of costing of service delivery studies had been carried out. Methodologically, this current study was similar to other costing studies though the other studies calculated the costs of services such as, pregnancy-related healthcare, family planning program implementation in Bangladesh [2,4]. Hutchinson *et al.*[6] undertook a cost-effectiveness analysis of a national health communication program in rural Bangladesh in 2001–2003. In that study, cost for BCC intervention through Mass Media (like, radio, Billboard) was calculated for the Smiling Sun Health Communication program in Bangladesh for period 2001–2003. They found that the cost per Billboard for ANC related message delivery was US$ 823.48 or 45,291 BDT (1 US$ = 55 BDT in year 2003). The Billboard was estimated to be watched by 2,601 people on average each year, which means that the cost per exposed was 17.4 BDT (or 40.1 BDT at price level of 2011). In contrast, we found the cost per billboard exposure was 7.87 BDT. The smaller cost in our study can be partially explained by exposure to a higher number of people in urban slums compared to with lower exposure in the rural study area. In another cost-effectiveness study in the Philippines, Kincaid *et al.*[7] calculated the cost associated with a TV program on family planning. They found that the cost per person for TV exposure was $ 0.06 or 3.3 BDT (equivalent to 8.44 BDT at the price level of 2011) in year 2000–2001. We found a lower cost per exposed by TV-spot (0.39BDT) in *Manoshi* project. It may be partly explained by the different country context (both population density and expenses), as well as the differences in broadcasting, as the current project ran TV spot on a local cable TV operator, while the comparison project ran commercials on a national channel in the Philippines. Moreover, it was likely that higher numbers of people were exposed in highly populous urban slums in Bangladesh, compared to the Philippines.

## Conclusions

The cost per exposure varied substantially across the different BCC intervention channels. The inputs contributing the highest proportion of costs were personnel, BCC materials and refreshments. However, the major cost drivers differed across channels and sub-channels. The BCC costs of sub-channels can be useful for planning and predicting the total cost of BCC interventions in current and future projects. In future, this information might be used to plan resource allocation between sub-channels by comparing the cost in relation to the impact (measured by increased knowledge and better practice of MNCH program). In that case, cost-effectiveness analysis needs to be performed. It is important to note that sub-channels may be substitutive and complementary which should be assessed by analyzing the content of each sub-channel.

## Abbreviations

BRAC: Bangladesh rural advancement committee; MDG: Millennium development goals; MNCH: Maternal neonatal and child health; BCC: Behavior change communication; EDD: Expected date of delivery; ANC: Antenatal care; PNC: Post-natal care; SS: Shasthya Sebika (community health worker); SK: Shasthya Karmi (community health worker); PO: Program organizers; MMW: Manoshi mid-wife; BM: Branch manager; FFC: Face-to-face counseling; GC: Group counseling; MM: Mass media; WSG: Women support group.

## Competing interests

The authors declare that they have no competing interests.

## Authors’ contributions

BKS and JAMK conceptualize the research idea and study design as well as contributed to data analysis, literature review, writing and revision of the manuscript. SA contributed to literature review data analysis, writing, revising and finalizing the manuscript. NI contributed to literature review, data analysis, writing and critically revised the manuscript. All authors read and approved the manuscript before submission.
